# UDP-glucuronosyltransferase 1A1*6 and *28 polymorphisms as indicators of initial dose level of irinotecan to reduce risk of neutropenia in patients receiving FOLFIRI for colorectal cancer

**DOI:** 10.1007/s10147-015-0937-x

**Published:** 2015-12-28

**Authors:** Yoshinori Miyata, Tetsuo Touyama, Takaya Kusumi, Yoshitaka Morita, Nobuyuki Mizunuma, Fumihiro Taniguchi, Mitsuaki Manabe

**Affiliations:** 1 Department of Medical Oncology, Saku Central Hospital Advanced Care Center, 3400-28 Nakagomi, Saku, Nagano 385-0051 Japan; 2 Department of Surgery, Nakagami Hospital, Okinawa, Japan; 3 Department of Surgery, Keiyukai Sapporo Hospital, Sapporo, Japan; 4 Department of Radiology, National Hospital Organization Kobe Medical Center, Kobe, Japan; 5 Department of Gastroenterology, Cancer Institute Hospital of Japanese Foundation for Cancer Research, Tokyo, Japan; 6 Hepato-Pancreatic Surgery Division, Japanese Red Cross Kyoto Daiichi Hospital, Kyoto, Japan; 7 Yakult Honsha Co., Ltd., Tokyo, Japan

**Keywords:** Colorectal, Irinotecan, Neutropenia, Polymorphism, UGT1A1 enzyme

## Abstract

**Background:**

Irinotecan (CPT-11)-induced neutropenia is associated with *UDP-glucuronosyltransferase* (UGT) 1A1*6 and *28 polymorphisms. This prospective study investigated whether using these polymorphisms to adjust the initial dose of CPT-11 as part of FOLFIRI treatment in colorectal cancer patients might improve safety.

**Methods:**

All data were collected by a physician. The relationship between *UGT1A1* polymorphisms and first-cycle neutropenia, reasons for treatment discontinuation, and time-to-treatment failure were evaluated. Multivariate analysis was used to assess the risk of neutropenia.

**Results:**

A total of 795 patients were divided into wild-type (*1/*1) (50.1 %), heterozygous (*28/*1, *6/*1) (41.1 %), and homozygous (*28/*28, *6/*6, *28/*6) (8.which are associated with a decrease in the8 %) groups, in which the median starting dose of CPT-11 was 143.0, 143.0, and 115.0 mg/m^2^, respectively. First-cycle grade ≥3 neutropenia occurred in 17.3, 25.4, and 28.6 % of these patients, respectively. Multivariate analysis revealed that the incidence of grade ≥3 neutropenia was significantly greater in the heterozygous and homozygous groups than in the wild-type group [odds ratio (OR) 1.67; 95 % confidence interval (CI) 1.16–2.42; *p* = 0.0060, and OR 2.22; 95 % CI 1.22–4.02; *p* = 0.0088, respectively]. Age (OR 1.77; 95 % CI 1.24–2.53; *p* = 0.0017), coelomic fluid (OR 1.84; 95 % CI 1.05–3.25; *p* = 0.0343), and non-reduction in starting dose (OR 1.53; 95 % CI 1.08–2.18; *p* = 0.0176) were also identified as significant risk factors.

**Conclusion:**

The risk of neutropenia was higher in the heterozygous and homozygous groups at initiation of CPT-11 treatment. This suggests that when a reduction in dose is required in patients harboring two variant alleles, the decrease should be approximately 20 %.

## Introduction

Irinotecan hydrochloride hydrate (CPT-11), a derivative of the antitumor alkaloid camptothecin, inhibits topoisomerase I, and is used to treat various types of tumor, including those arising as gastroenterological, lung, or gynecological cancers. As such, it forms part of one of the standard chemotherapy regimens for colorectal cancer, FOLFIRI, where it is used in combination with 5-fluorouracil (5-FU) and l-leucovorin (l-LV) [1, 2].

Irinotecan is hydrolyzed to its active metabolite, SN-38, by carboxylesterase, primarily in the human liver [3, 4]. SN-38 is then metabolized to a non-toxic glucuronide, SN-38G, by UDP-glucuronosyltransferase (UGT) 1A1, a molecular species of UGT in the liver. This metabolite is primarily excreted into the bile and transferred to the intestine [5, 6]. Genetic polymorphisms of UGT1A1 include UGT1A1*28 and UGT1A1*6, which are associated with a decrease in the formation of SN-38G, and thus delayed metabolism of SN-38 in the order wild-type, heterozygous, and homozygous. As a result, patients with *UGT1A1* polymorphisms are more susceptible to toxicities such as neutropenia [7–9].

In 2005, the US Food and Drug Administration revised the Dosage and Administration section on the labeling of CPT-11 as follows: “When administered in combination with other agents, or as a single-agent, a reduction in the starting dose by at least one level of CAMPTOSAR (brand name of CPT-11) should be considered for patients known to be homozygous for the UGT1A1*28 allele. However, the precise dose reduction in this patient population is not known”.

The allele frequency of UGT1A1*28 is lower in Asians (8.6–13.0 % in Japanese) than in Caucasians (29.5–38.8 %). In contrast, although the frequency of the UGT1A1*6 allele is very low in Caucasians, it is relatively common in Asians (13.0–17.7 % in Japanese) [10], which partly contributes to an increased risk of CPT-11-induced toxicity in this group. Therefore, in Japan, some studies have investigated the relationship between *UGT1A1* genetic polymorphisms and risk, focusing on the following three groups—*1/*1 (wild-type group); *28/*1 and *6/*1 (heterozygous group); and *28/*28, *6/*6, and *28/*6 (homozygous group). Double heterozygosity (*28/*6) was classified as homozygous based on the results of other earlier studies [7, 11, 12]. The results of one of these earlier clinical studies were inconclusive as to the optimum dose of CPT-11 for a homozygous group [11].

Approval for testing for UGT1A1*6 and *28 genetic polymorphisms using the Invader UGT1A1 Molecular Assay (Sekisui Medical Co., Ltd, Tokyo, Japan) under national insurance was finally given in November 2008 in Japan. The test kit then became available in March 2009, making this an easy and viable part of therapy in a clinical setting.

The purpose of this prospective study was to investigate whether UGT1A1*6 and *28 polymorphisms could be used to determine the initial dose level of CPT-11 to improve safety in patients receiving FOLFIRI for colorectal cancer.

## Patients and methods

### Patients

Patients were eligible for inclusion in the present study if they met all of the following criteria—CPT-11-naive; no evidence of myelosuppression, infectious disease, diarrhea (watery stool), intestinal paralysis or obstruction, ascites, or jaundice; a diagnosis of colorectal cancer; scheduled to receive FOLFIRI; and provided informed consent to participate in this study. This study was conducted in accordance with Good Post-marketing Study Practice (GPSP) of the Ministry of Health, Labour and Welfare, Japan. Approval by the Institutional Review Board of each institution was not mandatory, as the GPSP does not require such approval for post-marketing surveillance.

### Classification of *UGT1A1* polymorphisms

Three groups were established based on the results of testing for UGT1A1*6 and *28 genetic polymorphisms—wild-type group (*1/*1); heterozygous group (*28/*1 and *6/*1); and homozygous group (*28/*28, *6/*6, and *28/*6). Double heterozygosity (*28/*6) was classified as homozygous based on the results of previous studies [7, 11, 12].

### Treatment

FOLFIRI was administered according to a previously reported standard schedule in Japan—CPT-11, 5-FU (bolus), 5-FU (infusion), and l-LV at 150, 400, 2,400, and 200 mg/m^2^, respectively [2, 13]. Because this was a non-interventional study, the dose level and dosing interval were modified at the discretion of the primary physician. In this study, a dose reduction was defined as 5 % lower (142.5 mg/m^2^) than the standard dose in Japan (150 mg/m^2^). Information on treatment days and dosing levels was collected for 1 year after the start of treatment. Time-to-treatment failure (TTF) was defined as the period of time from the start of treatment until the end of treatment due to ‘progressive disease or tumor death’, ‘adverse event-related (side-effect) or treatment-related death’, or ‘withdrawal from FOLFIRI’.

### Safety evaluation

Adverse events were evaluated according to the Common Terminology Criteria for Adverse Events v3.0. The follow-up period was defined as 1 year after the start of treatment.

### Statistical analyses

All analyses were performed using SAS software version 9.2 (SAS Institute, Cary, NC, USA). TTF according to *UGT1A1* genetic polymorphisms was assessed using Kaplan–Meier curves, and significance was determined using the log-rank test. Using the first cycle as the target, the incidence of grade ≥3 neutropenia was analyzed in a multivariate model using a step-down procedure to select variables; significance level was set at *p* = 0.1. The variables selected were genetic polymorphism, age, sex, Eastern Cooperative Oncology Group performance status (PS), prior chemotherapy, new onset/recurrence, coelomic fluid, complications, and starting dose reduction. Differences were considered statistically significant when the two-tailed *p* value was <0.05.

## Results

### Baseline characteristics of patients

**Fig. 1:**
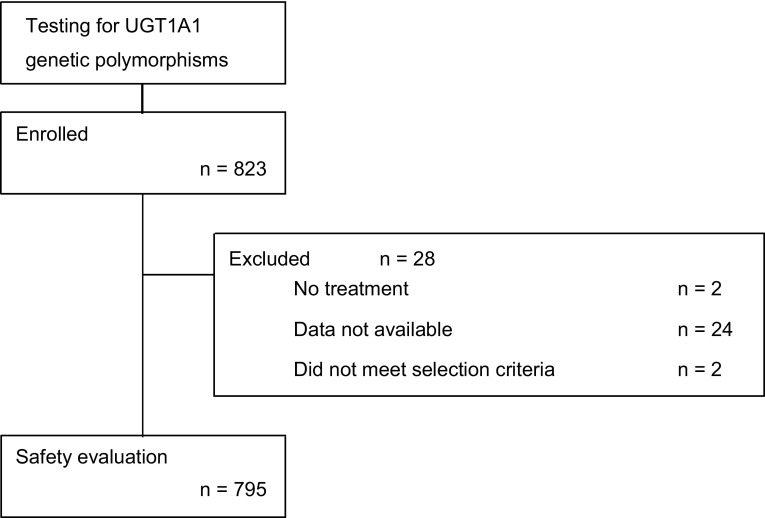
Flow chart. A total of 823 patients tested for *UGT1A1* genetic polymorphisms were enrolled in the study. Overall, 795 patients were included in safety evaluation after excluding 28 patients who did not meet the inclusion criteria

A total of 823 patients from 210 institutions were initially enrolled in the study between April 2009 and March 2011. Of these, 795 patients were included in the safety evaluation after excluding two patients not treated with CPT-11, 24 for whom no data were available, and two who did not meet the selection criteria (Fig. 1). Of the 795 patients evaluated, 398 (50.1 %) were wild-type, 327 (41.1 %) were heterozygous, and 70 (8.8 %) were homozygous according to UGT1A1*6 and *28 polymorphisms (Table 1). The median ages of the patients in the wild-type, heterozygous, and homozygous groups were 67, 66, and 65 years, respectively; 151 (37.9 %), 112 (34.3 %), and 20 (28.6 %) patients were aged ≥70 years in each group, respectively. No marked imbalance was noted in any other baseline characteristics (Table 2).

**Table 1:** Frequencies of *UGT1A1* genotype in this study

*UGT1A1* genotype	*n*	(%)
All patients	795	
Wild-type group	398	(50.1)
*1/*1	398	(50.1)
Heterozygous group	327	(41.1)
*6/*1	195	(24.6)
*28/*1	132	(16.6)
Homozygous group	70	(8.8)
*6/*6	14	(1.8)
*28/*28	12	(1.5)
*28/*6	44	(5.5)

*UGT* UDP-glucuronosyltransferase

**Table 2:** Baseline characteristics of patients

Characteristics	Wild-type (*n* = 398), *n* (%)	Heterozygous (*n* = 327), *n* (%)	Homozygous (*n* = 70), *n* (%)
Age (years)			
Median	67.0	66.0	65.0
Range	29–86	35–86	36–81
<70	247 (62.1)	215 (65.7)	50 (71.4)
≥70	151 (37.9)	112 (34.3)	20 (28.6)
Sex			
Male	234 (58.8)	205 (62.7)	41 (58.6)
Female	164 (41.2)	122 (37.3)	29 (41.4)
Performance status			
0	298 (74.9)	242 (74.0)	48 (68.6)
1	88 (22.1)	72 (22.0)	22 (31.4)
≥2	12 (3.0)	13 (4.0)	0 (−)
Prior chemotherapy			
Absent	73 (18.3)	57 (17.4)	12 (17.1)
Present	325 (81.7)	270 (82.6)	58 (82.9)
New onset/recurrent			
New onset	201 (50.5)	174 (53.2)	33 (47.1)
Recurrent	197 (49.5)	153 (46.8)	37 (52.9)
Coelomic fluid			
Absent	374 (94.0)	295 (90.2)	65 (92.9)
Present	24 (6.0)	32 (9.8)	5 (7.1)
Complications			
Absent	250 (62.8)	193 (59.0)	48 (68.6)
Present	148 (37.2)	134 (41.0)	22 (31.4)

### CPT-11 administration

**Fig. 2:**
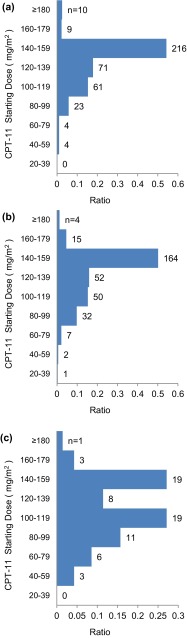
Distribution of starting dose of CPT-11 in **a** wild-type (*n* = 398), **b** heterozygous (*n* = 327), and **c** homozygous groups (*n* = 70)

Table 3 shows the starting doses of each drug administered as part of FOLFIRI. The dose level of 5-FU did not differ among the three groups, but the median starting doses of CPT-11 were 143.0, 143.0, and 115.0 mg/m^2^ in the wild-type, heterozygous, and homozygous groups, respectively (Table 3). A total of 204 patients (50.1 %) in the wild-type group, 164 (51.3 %) in the heterozygous group, and 23 (32.9 %) in the homozygous group received a starting dose of CPT-11 of ≥142.5 mg/m^2^. A bimodal distribution was observed in the homozygous group, but not in the wild-type or heterozygous groups (Fig. 2).

**Table 3:** State of clinical use

	Wild-type (*n* = 398)	Heterozygous (*n* = 327)	Homozygous (*n* = 70)
Starting dose (mg/m^2^)			
CPT-11, median (range)	143.0 (56–185)	143.0 (23–181)	115.0 (41–180)
Bolus 5-FU, median (range)	384.0 (0–796)	380.0 (0–800)	383.5 (0–517)
Infusional 5-FU, median (range)	2327.0 (538–2542)	2312.0 (178–2830)	2299.0 (0–2469)
Distribution of starting CPT-11 dose			
<142.5 mg/m^2^ (%)	194 (48.7)	163 (49.8)	47 (67.1)
≥142.5 mg/m^2^ (%)	204 (51.3)	164 (50.2)	23 (32.9)

*CPT*-*11* irinotecan, *5*-*FU* 5-fluorouracil

### Safety

The incidence of grade ≥3 neutropenia in the first cycle tended to increase in the order wild-type < heterozygous < homozygous (17.3, 25.4, 28.6 %, respectively), and this tendency persisted throughout the treatment (44.7, 54.1, and 57.1 %, respectively) (Table 4). The percentages of patients withdrawn from treatment were similar for all *UGT1A1* genotypes (Table 5).

**Table 4:** Median TTF and reasons for treatment discontinuation

	Wild-type (*n* = 398)	Heterozygous (*n* = 327)	Homozygous (*n* = 70)
TTF			
Median	161.5	165.0	136.0
95 % CI	142.0–183.0	148.0–177.0	106.0–177.0
Reasons for discontinuing FOLFIRI			
Progressive disease (%)	183 (46.0)	169 (51.7)	33 (47.1)
Adverse events (%)	59 (14.8)	52 (15.9)	10 (14.3)
Withdrawal of FOLFIRI (%)	75 (18.8)	53 (16.2)	14 (20.0)

*TTF* time-to-treatment failure, *CI* confidence interval, *FOLFIRI* l-leucovorin, 5-fluorouracil, and irinotecan

**Table 5:** Association between *UGT1A1* genotype and irinotecan toxicities

Toxicities	Wild-type (*n* = 398), *n* (%)	Heterozygous (*n* = 327), *n* (%)	Homozygous (*n* = 70), *n* (%)
First-cycle neutropenia			
Grade ≥1	209 (52.5)	199 (60.9)	42 (60.0)
Grade ≥3	69 (17.3)	83 (25.4)	20 (28.6)
Neutropenia			
Grade ≥1	311 (78.1)	266 (81.3)	55 (78.6)
Grade ≥3	178 (44.7)	177 (54.1)	40 (57.1)
All adverse events			
Grade ≥1	383 (96.2)	321 (98.2)	68 (97.1)
Grade ≥3	229 (57.5)	223 (68.2)	48 (68.6)

Multivariate analysis was performed to identify risk factors for first-cycle grade ≥3 neutropenia. Heterozygosity and homozygosity were identified as significant risk factors compared with wild-type [heterozygous group: *p* = 0.0060; odds ratio (OR) 1.67; 95 % confidence interval (CI) 1.16–2.42, and homozygous group: *p* = 0.0088; OR 2.22; 95 % CI 1.22–4.02]. Age ≥70 years (*p* = 0.0017; OR 1.77; 95 % CI 1.24–2.53), coelomic fluid (*p* = 0.0343; OR 1.84; 95 % CI 1.05–3.25), and non-reduction in starting dose (*p* = 0.0176; OR 1.53; 95 % CI 1.08–2.18) were also identified as independent significant risk factors (Table 6).

**Table 6:** Multivariate predictors of treatment-related grade ≥3 neutropenia

	Neutropenia		
	OR	(95 % CI)	*p* value*
*UGT1A1* genotype (wild-type vs heterozygous)	1.67	1.16–2.42	0.0060
*UGT1A1* genotype (wild-type vs homozygous)	2.22	1.22–4.02	0.0088
Age (years) (<70 vs ≥70)	1.77	1.24–2.53	0.0017
Sex (male vs female)	1.38	0.97–1.95	0.0726
Coelomic fluid (absent vs present)	1.84	1.05–3.25	0.0343
Starting dose reduction (reduction vs non-reduction)	1.53	1.08–2.18	0.0176

*UGT* UDP-glucuronosyltransferase, *OR* odds ratio, *CI* confidence interval

* Chi-squared test

### Efficacy

**Fig. 3:**
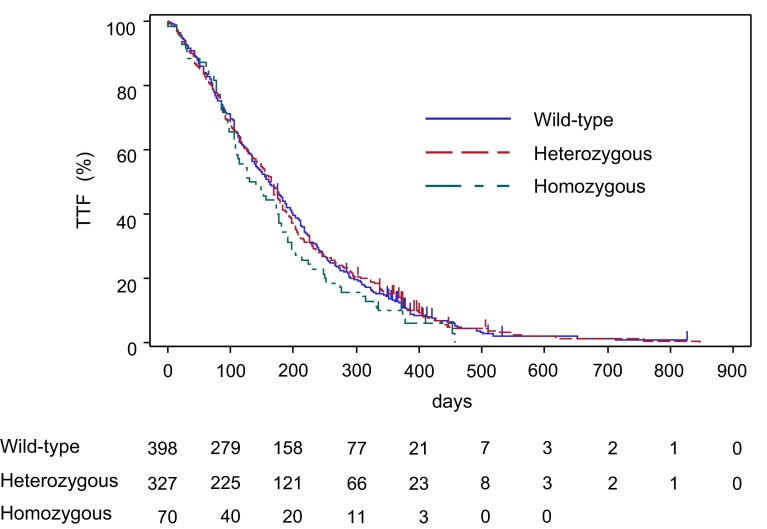
Kaplan–Meier curves for time from start of treatment to discontinuation of treatment according to *UGT1A1* genetic polymorphisms. Patients not withdrawn from treatment during the follow-up period (1 year after start of treatment) were censored on the last dosing day of the final treatment cycle

Figure 3 shows the Kaplan–Meier curves obtained for TTF according to *UGT1A1* genetic polymorphism. No significant difference was observed among the *UGT1A1* genetic polymorphisms [wild-type group vs heterozygous or homozygous group: *p* = 0.7390; hazard ratio (HR) 1.025; 95 % CI 0.888–1.183, and wild-type or heterozygous group vs homozygous group: *p* = 0.1582; HR 1.197; 95 % CI 0.931–1.540]. The median TTF was 161.5 (95 % CI 142.0–183.0), 165.0 (95 % CI 148.0–177.0), and 136.0 (95 % CI 106.0–177.0) days in the wild-type, heterozygous, and homozygous groups, respectively (Table 4).

The present results indicated little difference in effect of each polymorphism on TTF, regardless of starting dose (data not shown).

## Discussion

The goal of the present study was to investigate whether UGT1A1*6 and *28 polymorphisms could be used to determine the initial dose level of CPT-11 to improve safety in patients receiving FOLFIRI for colorectal cancer. These results comprise the largest published prospective analysis to date.

The multivariate analysis revealed that the incidence of neutropenia increased significantly in those groups harboring one or more alleles compared with the wild-type group—the heterozygous and homozygous groups showed a 1.67-fold and 2.22-fold increase in risk of grade ≥3 neutropenia, respectively. Minami et al. reported that neutropenia was associated with homozygosity for UGT1A1*6 or *28 [9]. However, Hoskins et al. [14] and Hu et al. [15] reported inconsistent results from meta-analyses of the relationship between homozygosity for UGT1A1*28 and neutropenia in patients treated with CPT-11 at a starting dose of <150 mg/m^2^, and no definitive relationship was therefore demonstrated. The results of the present study, albeit non-interventional, support the findings of Minami et al. and Hu et al. Moreover, the present study was based on an analysis of data from a single set of 795 patients, and therefore comprises a larger sample size than those in the above-mentioned studies. Satho et al. [11] and Hazama et al. [16] found that the pharmacokinetics of SN 38 were higher in patients harboring one variant allele than in those who were not. Moreover, in the Satho study, there was a significant correlation between an increase in the rate of severe neutropenia and an increase in the pharmacokinetics of SN38. Taken together, these results suggest that the initial dose of CPT-11 should be considered with care in patients homozygous or heterozygous for *UGT1A1* in clinical practice in Japan. However, previous studies reported that the risk for those who were heterozygous was small, indicating the need for further evaluation of clinical information on UGT1A1*6 and *28 heterozygous groups in future studies.

The observed OR for the risk of neutropenia in the homozygous group compared with the wild-type group (2.22) was lower than (5.21–8.63) reported in previous studies [11, 17]. Hoskins et al. [14] reported that the risk of neutropenia abated with a decrease in the dose of CPT-11. In their study, the OR was 1.80 at lower doses, which represented no significant difference between homozygous and wild-type or heterozygous individuals, similar to the findings in the present study.

In addition to *UGT1A1* polymorphisms, the present study also identified age ≥70 years, coelomic fluid, and non-reduction in starting dose as risk factors for grade ≥3 neutropenia. The relationship between age and risk of neutropenia remains unexplained, although older patients are generally more likely to experience adverse events, probably because of age-related changes in pharmacokinetics or pharmacodynamics and an increased prevalence of chronic diseases. The trend observed in the present study is in line with the results of an earlier study showing that CPT-11 monotherapy was associated with a higher risk of grade ≥3 neutropenia in the elderly [18]. The relationship between coelomic fluid and risk of neutropenia also remains poorly understood, although an increased risk of CPT-11-related leukopenia in pleural effusion and massive ascites was reported [19]. We believe that the retention of coelomic fluid decreases intestinal peristalsis, thereby delaying excretion of CPT-11 and increasing toxicity. In addition, there appears to be an association between coelomic fluid and peritoneal dissemination. This suggests that the disease is likely to be at a more advanced stage by the time treatment is initiated in patients with coelomic fluid. Taken together with the results from earlier studies, the present results suggest that patient age and presence of coelomic fluid should also be taken into consideration when planning treatment in a clinical setting.

In the present study, the starting dose of CPT-11 was reduced in approximately 67 % of patients in the homozygous group, but not in the wild-type or heterozygous groups, resulting in a median starting dose that was approximately 20 % lower in the homozygous group. A previous study [11] investigating dose levels in Japanese patients according to UGT1A1*6 and *28 polymorphisms demonstrated the safety of 150 mg/m^2^ in wild-type and heterozygous groups; however, no recommended dose was identified for homozygous patients because of a large individual variation in pharmacokinetics.

The results of the present study indicate that testing for UGT1A1*6 and *28 genetic polymorphisms could be useful in determining the appropriate starting dose of CPT-11 in clinical practice.

An analysis of treatment duration in relation to *UGT1A1* genetic polymorphisms revealed that TTF was similar between the wild-type, heterozygous, and homozygous groups, as were the reasons for treatment discontinuation. Blood drug concentrations were not measured in the current study, but the metabolism of SN-38 is delayed in individuals homozygous for *UGT1A1* polymorphisms [9, 20]. In the present study, the dose level in the homozygous group was reduced, allowing the pharmacokinetics of SN-38 to be maintained without compromising its antitumor efficacy and avoiding serious adverse events requiring treatment discontinuation; therefore, there was no effect on the treatment duration. However, caution should be taken in reducing the dose, as it may increase the risk of ineffective treatment.

In conclusion, the present results revealed that patients harboring one or more alleles had a higher risk of neutropenia at initiation of treatment, indicating the importance of testing for *UGT1A1* genetic polymorphisms before commencing therapy. These results also suggest that when a reduction in dose is required in patients harboring two variant alleles, the decrease should be approximately 20 %.
